# Dual Therapy Treatment Strategies for the Management of Patients Infected with HIV: A Systematic Review of Current Evidence in ARV-Naive or ARV-Experienced, Virologically Suppressed Patients

**DOI:** 10.1371/journal.pone.0148231

**Published:** 2016-02-05

**Authors:** Jean-Guy Baril, Jonathan B. Angel, M. John Gill, Joseph Gathe, Pedro Cahn, Jean van Wyk, Sharon Walmsley

**Affiliations:** 1 Clinique médicale du Quartier latin, Montreal, Quebec, Canada; 2 Division of Infectious Diseases, University of Ottawa and the Ottawa Hospital, Ottawa, Ontario, Canada; 3 Department of Medicine, University of Calgary, Calgary, Alberta, Canada; 4 Therapeutic Concepts, Houston, Texas, United States of America; 5 Fundación Huesped, Buenos Aires, Argentina; 6 AbbVie Inc., North Chicago, Illinois, United States of America; 7 Toronto General Research Institute, University Health Network, Toronto, Ontario, Canada; 8 University of Toronto, Toronto, Ontario, Canada; Imperial College London, UNITED KINGDOM

## Abstract

**Objective:**

We reviewed the current literature regarding antiretroviral (ARV)-sparing therapy strategies to determine whether these novel regimens can be considered appropriate alternatives to standard regimens for the initial treatment of ARV-naive patients or as switch therapy for those patients with virologically suppressed HIV infection.

**Methods:**

A search for studies related to HIV dual therapy published from January 2000 through April 2014 was performed using Biosis, Derwent Drug File, Embase, International Pharmaceutical Abstracts, Medline, Pascal, SciSearch, and TOXNET databases; seven major trial registries, and the abstracts of major conferences. Using predetermined criteria for inclusion, an expert review committee critically reviewed and qualitatively evaluated all identified trials for efficacy and safety results and potential limitations.

**Results:**

Sixteen studies of dual therapy regimens were critiqued for the ARV-naive population. Studies of a protease inhibitor/ritonavir in combination with the integrase inhibitor raltegravir or the nucleoside reverse transcriptase inhibitor lamivudine provided the most definitive evidence supporting a role for dual therapy. In particular, lopinavir/ritonavir or darunavir/ritonavir combined with raltegravir and lopinavir/ritonavir combined with lamivudine demonstrated noninferiority to standard of care triple therapy after 48 weeks of treatment. Thirteen trials were critiqued in ARV-experienced, virologically suppressed patients. The virologic efficacy outcomes were mixed. Although overall data regarding toxicity are limited, when compared with standard triple therapy, certain dual therapy regimens may offer advantages in renal function, bone mineral density, and limb fat changes; however, some dual combinations may elevate lipid or bilirubin levels.

**Conclusions:**

The potential benefits of dual therapy regimens include reduced toxicity, improved tolerability and adherence, and reduced cost. Although the data reviewed here provide valuable insights into the effectiveness and tolerability of dual therapy regimens, it remains unclear whether these potential benefits can be maintained long-term. Appropriately powered studies with longer follow-up periods are needed to more definitively assess potential toxicity reduction advantages with dual therapy.

## Introduction

In the late 1980s/early 1990s, the sequential use of nucleoside reverse transcriptase inhibitor (NRTI) monotherapy and dual therapies in patients with HIV infection rapidly led to treatment failure because of the emergence of resistance-associated mutations [1]. The use of combination antiretroviral therapy (cART) began in the mid-1990s, in which 2 NRTIs were combined with a third agent from a different therapeutic class. Current treatment guidelines continue the convention of preferred cART based on combining a dual NRTI backbone with a third “anchor” agent, such as a ritonavir (r)-boosted protease inhibitor (PI; PI/r), a non-nucleoside reverse transcriptase inhibitor (NNRTI), or an integrase inhibitor [2–4]. The toxicities associated with long-term use of NRTIs have led to the assessment of dual therapy approaches that do not include an NRTI component. A higher risk of treatment failure was observed in early NRTI-sparing studies compared with current standard triple therapy regimens [5–7]. Cohort studies suggest that patients with HIV are now living longer and are encountering an increased prevalence of comorbidities associated with natural aging, including renal, cardiovascular, or liver diseases; cognitive decline; metabolic disorders (diabetes and dyslipidaemia); and osteoporosis [8,9]. Drug-related adverse events (AEs) associated with the long-term use of antiretroviral therapy (ARV) may contribute to these comorbidities [10–13].

With the improved potency, tolerability, and durability of newer drugs and the higher barrier to the development of resistance, interest has re-emerged for ARV-sparing strategies, including monotherapy and dual therapies. These strategies have been applied as initial therapy in ARV-naive patients or as a switch strategy in those patients who have become virologically suppressed on standard regimens. Ideally, these regimens should achieve and maintain viral suppression and immunologic control while minimizing short- and long-term AEs, improve adherence and convenience, and reduce costs.

One well-studied therapeutic approach is the use of PI/r monotherapy following suppression with standard triple therapy. Although successful for a majority of patients, PI monotherapy was found to be associated with a statistically significant increased risk of virologic failure and an increased incidence of PI-associated resistance [14]. Although most failures were re-suppressed by reinitiating NRTI therapies, this strategy is reserved for special circumstances. Current guidelines do not include dual therapy regimens as a standard treatment strategy unless specific clinical characteristics (eg, comorbidities, pre-treatment viral load, and CD4 cell counts) of the individual patient warrant their use [2–4].

The objective of this report was to summarise data in the published literature regarding dual therapy approaches for treating ARV-naive patients and as a switch strategy for virologically suppressed patients on ARV therapy. We reviewed the literature from January 2000, with the approval of the first PI/r, until April 2014, in order to evaluate the efficacy of dual therapy regimens and the on long-term safety, AEs, and comorbidities associated with these regimens.

## Methods

PRISMA guidelines for reporting systematic reviews were followed. The checklist is available as dreorting information (S1 PRISMA Checklist).

### Search Strategy

ProQuest Dialog, Biosis, Derwent Drug File, Embase, International Pharmaceutical Abstracts, Medline, Pascal, and SciSearch databases were searched from January 2000 through April 2014 for studies related to HIV dual therapy. TOXNET was searched for AEs. Subject headings and keywords were tailored for each electronic resource using the following concepts: (atazanavir OR darunavir OR dolutegravir OR fosamprenavir OR indinavir OR lopinavir OR saquinavir) AND (efavirenz OR enfuvirtide OR etravirine OR lamivudine OR maraviroc OR nevirapine OR raltegravir OR rilpivirine OR saquinavir OR tenofovir OR tipranavir). The term “HIV dual therapy” was searched separately to capture potential combinations not explicitly stated above. The CRD/Cochrane Highly Sensitive Search Strategy [15] was used to restrict the research to randomised controlled trials in PubMed. For conference proceedings, we searched NLM Gateway (2008–2010); International AIDS Society Conference on HIV Pathogenesis and Treatment and Prevention (WAC/IAS) 2009–2013; Conference on Retroviruses and Opportunistic Infections (CROI) 2009–2014; International Congress on Drug Therapy in HIV Infection 2008, 2010, and 2012 (JIAS); International Workshop on Adverse Drug Reactions and Co-Morbidities in HIV 2009–2012; European AIDS Clinical Society (EACS) 2009, 2011, and 2013; and the Interscience Conference on Antimicrobial Agents and Chemotherapy (ICAAC) 2009–2013. Data were extracted from published abstracts or posters and oral presentations, where available. The following trial registries were searched for ongoing studies: Citeline’s TrialTrove, ClinicalTrials.gov, EuDRA, ANZCTR, Nederlands Trial Register, International Clinical Trials Registry Platform (ICTRP), and the International Federation of Pharmaceutical Manufacturers & Associations. We reviewed all identified trials and determined their suitability for inclusion.

### Eligibility Criteria

Eligible studies included randomised controlled or prospectively designed trials evaluating dual therapy combinations with a protease inhibitor (with or without r boosting [PI/r and PI, respectively]), an integrase inhibitor, an NNRTI, a CCR5 inhibitor, or lamivudine. A minimum 24-week duration of treatment in adults with HIV-1 infection who were ARV-naive or were switched after being virologically suppressed was required. Pilot/proof-of-concept studies and studies presented only as abstracts, which may not have been adequately powered, were also considered for inclusion. Case reports, reviews, correspondence, and research letters were excluded, as were phase 1, laboratory, pharmacokinetic/pharmacodynamic, prevention of vertical transmission, and retrospective studies or those including patients who were ARV-experienced but not suppressed, pediatric, or pregnant or patients who had a coinfection.

A primary outcome of suppression of viral load, change in viral load, or virologic failure (VF) was assessed; other outcomes were acceptable as a primary endpoint if they were supplemented by secondary endpoints that included the aforementioned criteria. Toxicity and/or comorbidity-related secondary outcomes were also evaluated.

### Data Abstraction and Qualitative Data Synthesis

The primary endpoint was efficacy (achieved or maintained virologic suppression, usually defined as <50 copies mL of HIV-1 RNA). Intent-to-treat (ITT) analyses were preferentially reported; however, per-protocol or observed analyses were also permitted. Changes in CD4 cell counts, discontinuation rates and tolerability or toxicity, lipid levels, renal function, bone mineral density (BMD), and body fat redistribution were examined, whether these parameters were predefined or reviewed post hoc. Subanalyses of included studies were also examined. In the trials, patients were classified as ARV-naive or virologically suppressed, and the results were examined qualitatively based on efficacy and safety results, as well as power and other study design limitations. The study entry criteria for inclusion, endpoints (actual rates and definitions), and comparators are presented in tabular format due to the large number of studies summarised in this review (n = 29).

## Results

**Fig 1:**
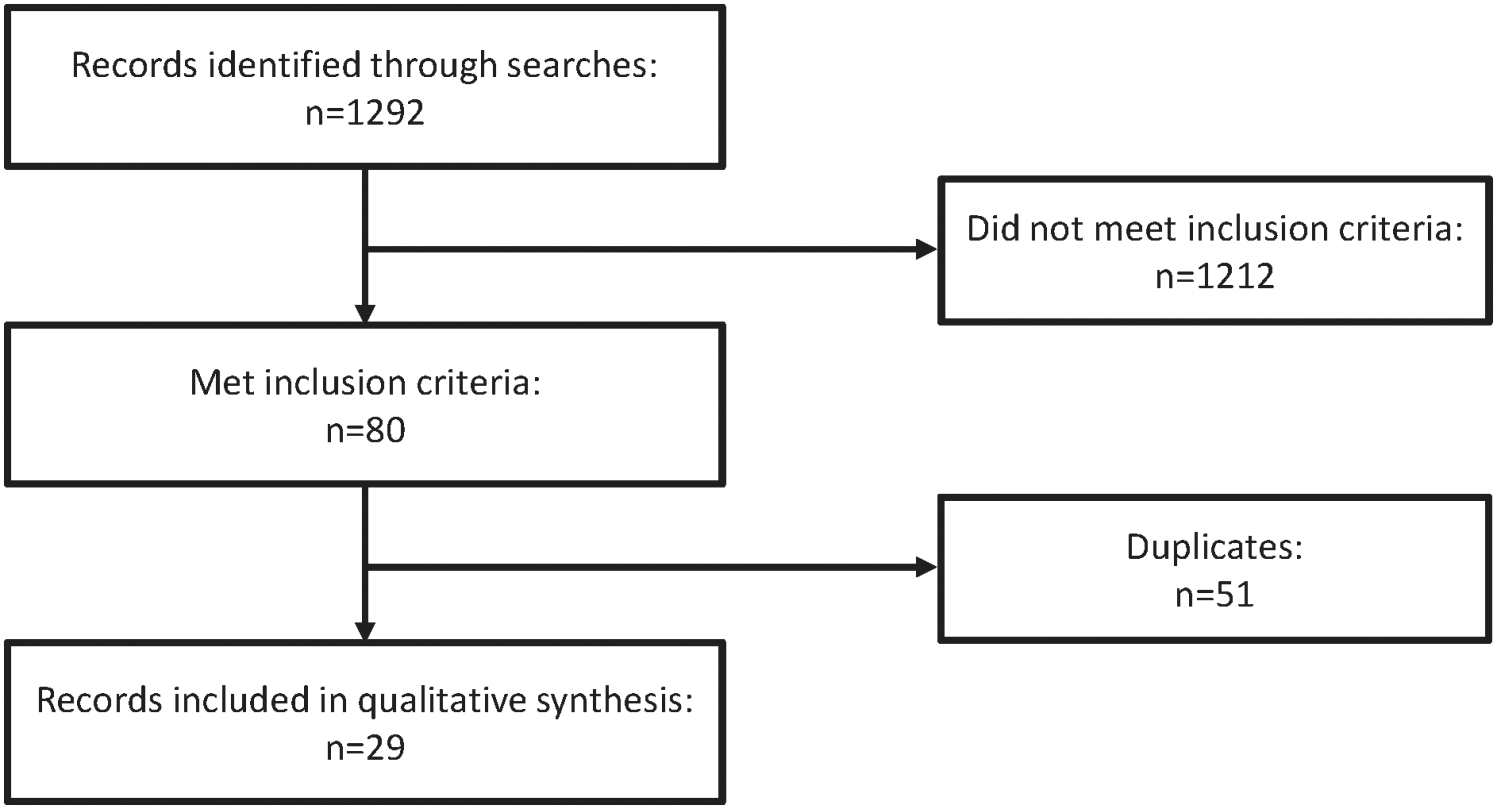
Flow diagram of literature search for systematic review.

Twenty-nine studies examining novel dual therapy regimens were included in the analysis (Fig 1); 16 trials contained ARV-naive patients and 13 had ARV-experienced, virologically suppressed patients.

### Trials in ARV-Naive Patients

**Fig 2:**
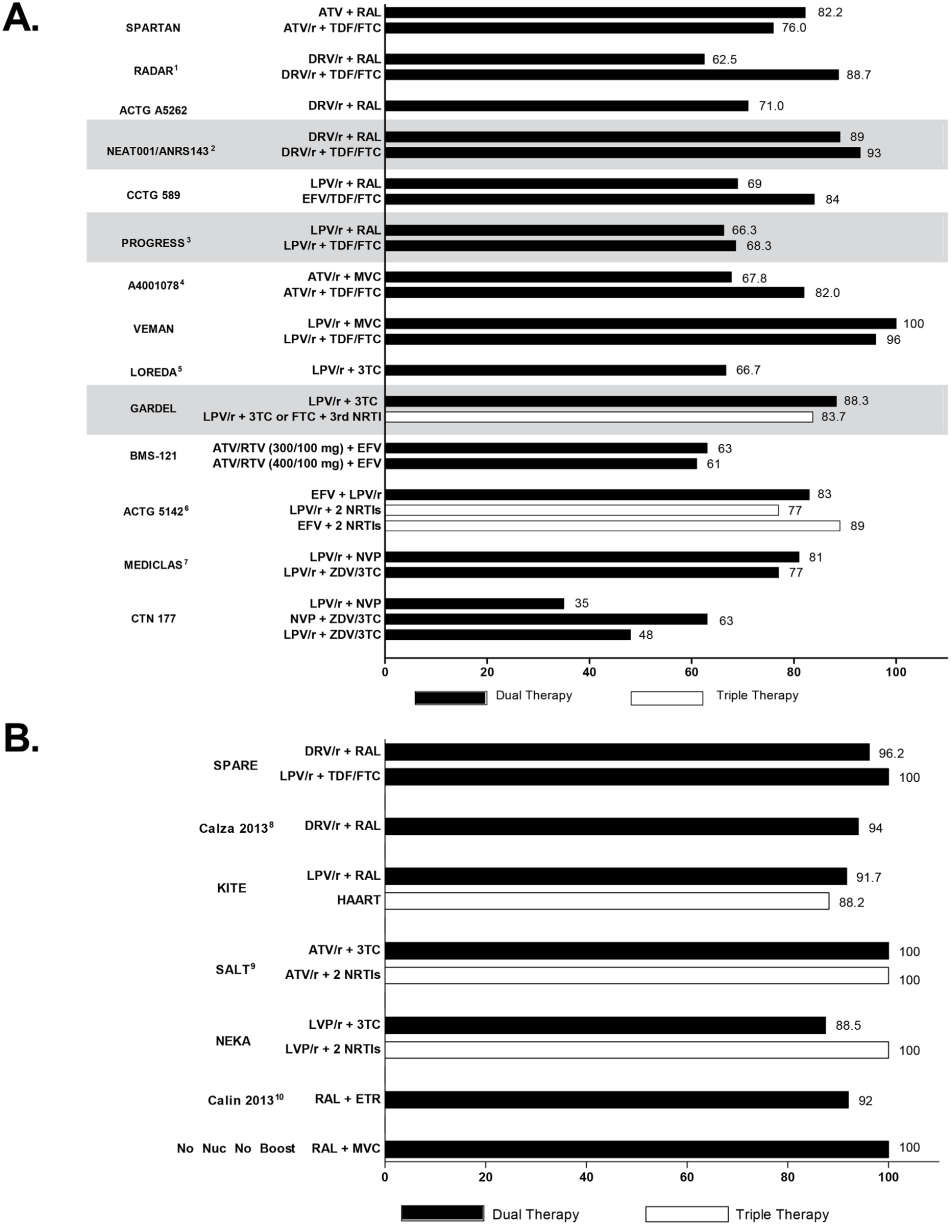
Efficacy of therapy by regimen in A) in ARV-naive, and B) ARV-experienced, virologically suppressed patients. Percentage indicated shows subjects with HIV-1 RNA <50 copies/mL at week 48. Studies that were randomised and sufficiently powered for direct comparison of standard and dual therapy regimens are shaded. ^1^<48 copies/mL; ^2^at 96 weeks; ^3^<40 copies/mL; ^4^at 96 weeks; ^5^<48 copies/mL; ^6^at 96 weeks; ^7^at 24 months; ^8^at 12 months; ^9^at week 24; ^10^<80 copies/mL. ARV, antiretroviral.

Sixteen trials of novel dual therapy regimens in ARV-naive patients were included; of these trials (Fig 1), many were underpowered to confirm noninferiority of the strategy relative to standard of care. Trial designs and key findings from these studies are summarised in Tables 1, 2 and 3 and are discussed briefly by strategy below. Virologic efficacy results are summarised in Fig 2A.

**Table 1:** Study designs for identified trials in ARV-naive patients.

Regimen	Study Name	Duration	Type	Treatment Arm	Dose	Primary Endpoint
**PI/r + RAL**	SPARTAN [16]	96 weeks (planned, terminated at 24 weeks but patients receiving treatment could continue)	Multicentre, randomised, open-label, non-comparative pilot study	ATV + RAL (n = 63, 45 evaluable at week 48)	300 mg BID + 400 mg BID	HIV-1 RNA <50 copies/mL at week 24 (ITT): 74.6% vs 63.3%
				ATV/r + TDF/FTC (n = 30, 25 evaluable at week 48)	300/100 mg QD + 300/200 mg QD	
	RADAR [17,18]	48 weeks, on-going	Randomised, open-label, pilot	DRV/r + RAL (n = 40)	800/100 mg QD + 400 mg BID	HIV-1 RNA <48 copies/mL at week 24 (ITT): 88.9% vs 81.0%
				DRV/r + TDF/FTC (n = 40)	800/100 mg QD +300/200 mg QD	
	ACTG A5262 [19]	52 weeks	Phase 2b, single-arm, open-label, multicentre	DRV/r + RAL (n = 112)	800/100 mg QD + 400 mg BID	VF by week 24 (ITT): 16% (17 patients)
	NEAT001/ANRS143 [20]	123 weeks	Phase 3, randomised, open-label, multicentre, parallel group	DRV/r + RAL (n = 401)	800/100 mg QD + 400 mg BID	Time to treatment failure (virologic or clinical) 17.4% vs 13.7%
				DRV/r + TDF/FTC (n = 404)	800/100 mg QD + 245/200 mg QD	
	CCTG 589 [21]	48 weeks	Randomised, open-label, pilot	LPV/r + RAL (n = 26)	Not reported	HIV-RNA <50 copies/mL, significantly higher with LPV/r + RAL at week 4 (*P* = 0.003) but not at week 48
				EFV/TDF/FTC (n = 25)	Not reported	
	PROGRESS [22–25]	96 weeks	Randomised, noninferiority, open-label, multicentre	LPV/r + RAL (n = 101)	400/100 mg BID + 400 mg BID	HIV-1 RNA <40 copies/mL at week 48 (ITT): 83.2% vs 84.8%
				LPV/r + TDF/FTC (n = 105)	400/100 mg BID + 300/200 mg QD	
**PI/r + MVC**	A4001078 [26,28,30]	48 weeks, later extended to 96 weeks	Phase 2b, randomised, open-label, pilot	ATV/r + MVC (n = 60)	300/100 mg QD + 150 mg QD	HIV-1 RNA <50 copies/mL at week 48 (ITT): 74.6% vs 83.6%
				ATV/r + TDF/FTC (n = 61)	300/100 mg QD + 300/200 mg QD	
	MIDAS [31]	48 weeks	Single-arm	DRV/r + MVC (n = 24)	800/100 mg QD + 150 mg QD	VF (HIV-1 RNA >50 copies/mL) at week 24 or later: 12.5% at week 24; 16.7% at week 48
	MODERN [32]	48 weeks	Interventional, randomised open-label	DRV/r + TDF/FTC (n = 406)	800/100 mg QD + 300/200 QD	Trial terminated after IDMC review due to inferior efficacy of MVC arm
				DRV/r + MVC (n = 406)	800/100 mg QD + 150 mg QD	
	VEMAN [27,29]	48 weeks	Prospective, randomised, open-label, proof-of-concept, multicentre	LPV/r + MVC (n = 26)	Not reported + 150 mg QD	HIV-1 RNA <50 copies/mL at week 48 (PP): 100% vs 96%
				LPV/r + TDF/FTC (n = 24)	Not reported	
**PI/r + 3TC**	LOREDA [33]	48 weeks	Phase 4, single-arm, multicentre, open-label, pilot	LPV/r + 3TC (n = 39)	400/100 mg BID + 300 mg QD	HIV-1 RNA <48 copies/mL at week 48 (ITT): 66.7%
	GARDEL [34]	48 weeks	Prospective, randomised, controlled, open label, noninferiority	LPV/r + 3TC (n = 217)	400/100 mg BID + 150 mg BID	HIV-1 RNA <50 copies/mL at week 48 (ITT): 88.3% (dual) vs 83.7% (triple)
				LPV/r + 3TC or FTC + third NRTI (n = 209)	400/100 mg BID + as appropriate	
**PI/r + NNRTI**	BMS-121 [35]	48 weeks	Randomised multicentre	ATV/r + EFV (n = 32)	300/100 mg QD + 600 mg QD	Mean percentage change from baseline in fasting plasma TG at week 8 in the combined treatment regimens: 61% (95% CI, 43.3%–80.7%)
				ATV/r + EFV (n = 33)	400/100 mg QD + 600 mg QD	
	ACTG 5142 [36,37]	Median follow-up of 112 weeks	Phase 3, randomised, multicenter, open-label	EFV + LPV/r (n = 250)	600 mg QD + 533/133 mg BID	Time to VF: significantly longer for EFV + 2 NRTIs vs LPV/r + 2 NRTIs; and time to regimen failure: no statistically significant differences between EFV + LPV/r and other groups in time to VF
				LPV/r + 2 NRTIs (n = 253)	400/100 mg BID + 2 NRTIs	
				EFV + 2 NRTIs (n = 250)	600 mg QD + 2 NRTIs	
	MEDICLAS [38]	24 months	Multicenter, multinational, single-blinded, randomised	LPV/r + NVP (n = 26)	533/133 mg + 200 mg BID	Changes in body composition and metabolic abnormalities. After 24 months, limb fat in the ZDV/3TC/LPV/r group was 1223±318 g lower than in the NVP/LPV/r group (*P* = 0.0002). At 24 months, 17/22 (77%) of the patients in the ZDV/3TC/LPV/r group and 21/26 (80%) in the NVP/LPV/r group had plasma HIV-RNA < 50 copies/mL
				LPV/r + ZDV/3TC (n = 22)	400/100 mg + 300/150 mg BID	
	CTN 177 [39]	96 weeks	Multicentre, randomised, prospective, open label	LPV/r + NVP (n = 26)	533/133 mg BID + 200 mg BID	Evaluate 48-week changes in mtDNA:nDNA ratio and efficacy, % (n) VL <50, ITT 48 weeks, safety, changes in metabolic parameters. LPV/r + NVP: −0.06, 42%; NVP + ZDV/3TC: −0.08, 50%; LPV/r + ZDV/3TC +0.26, 68%
				NVP + ZDV/3TC (n = 26)	200 mg BID + 300/150 mg	
				LPV/r + ZDV/3TC (n = 25)	400/100 mg BID + 300/150 mg BID	

3TC, lamivudine; ARV, antiretroviral; ATV, atazanavir; ATV/r, atazanavir/ritonavir; BID, twice a day; DRV/r, darunavir/ritonavir; EFV, efavirenz; IDMC = independent data monitoring company; ITT, intent-to-treat; LPV/r, lopinavir/ritonavir; MVC, maraviroc; NNRTI, non-nucleoside reverse transcriptase inhibitor; NRTI, nucleoside reverse transcriptase inhibitor; NVP, nevirapine, PI/r, ritonavir-boosted protease inhibitor; PP, per protocol; QD, once a day; r, ritonavir; RAL, raltegravir; TDF/FTC, tenofovir/emtricitabine; VF, virologic failure; ZDV, zidovudine.

**Table 2:** Results of key virologic endpoints from identified trials in ARV-naive patients^1^.

Regimen	Study Name	Treatment Arm	HIV-1 RNA <50 Copies/mL at Week 48 (Unless Specified)	Mean/ Median CD4 Increase (cells/mm^3^) at Week 48 (Unless Specified)	Discontinuations, n (%)	Treatment Failures/Virologic Failures^2^, n (%)	Mutations
**PI/r + RAL**	SPARTAN [16]	ATV + RAL (n = 63, 45 evaluable at week 48)	24 weeks: 74.6% (ITT); 48 weeks: 82.2% (OB: 37/45)	24 weeks: 166; 48 weeks: 235	6 (9.5%)	24 weeks VF: 11/63 (17.5%)	4 patients: Q148R (1), Q148Q/R and T97T/A (1), and N155H (2). No PI mutations
		ATV/r + TDF/FTC (n = 30, 25 evaluable at week 48)	24 weeks: 63.3% (ITT); 48 weeks: 76.0% (OB: 19/25)	24 weeks: 127; 48 weeks: 197	3 (10%)	24 weeks VF: 8/30 (26.7%)	None reported
	RADAR [17,18]	DRV/r + RAL (n = 40)	HIV-1 RNA<48 copies/mL; 24 weeks: 88.9%; 48 weeks: 62.5%	24 weeks: 123	3 (9%)	24 weeks VF: 2/39 (5.1%)	PI mutation: A71T (1); RAL testing pending
		DRV/r + TDF/FTC (n = 40)	HIV-1 RNA<48 copies/mL; 24 weeks: 81.0%; 48 weeks: 83.7%	24 weeks: 174	3 (9%)	24 weeks VF: 0/40 (0%)	None reported
	ACTG A5262 [19]	DRV/r + RAL (N = 112)	24 weeks: 79% (ITT); 48 weeks: 71% (ITT)	200	15 (13%)	24 weeks VF: 17/not reported (16%); 48 weeks VF: 28/not reported (26%)	5 of 25 tested: N155H (1), N155H/N (2), Q148Q/R and N155H/N (1), Q148K/Q and N155H/N (1). No PI mutations in 23 tested
	*NEAT 001/ANRS143 [20]*	*DRV/r + RAL (n = 401)*	*48 weeks*: *376/401*, *94%; 96 weeks*: *356/401*, *89%*	*96 weeks*: *267*	*Not reported*	*VF*: *17*.*4%*	*5/28; NRTI*, *1 (K65R); INI*, *5 (N155H)*. *No PI mutations*.
		*DRV/r + TDF/FTC (n = 404)*	*48 weeks*: *388/404*, *96%; 96 weeks*: *374/404*, *93%*	*96 weeks*: *266*	*Not reported*	*VF*: *13*.*7%*	*0/13*. *No mutations reported*.
	CCTG 589 [21]	LPV/r + RAL (n = 26)	69% (ITT); 86% (OB)	194	4 (15.4%)	No discontinuations due to VF	None reported
		EFV/TDF/FTC (n = 25)	84% (ITT); 88% (OB)	116	2 (8%)	No discontinuations due to VF	None reported
	*PROGRESS [22–25]*	*LPV/r + RAL (n = 101)*	*HIV-1 RNA <40 copies/mL*: *48 weeks*: *83*.*2% (ITT); 96 weeks*: *66*.*3% (ITT); 88*.*9% (OB)*	*48 weeks*: *215; 96 weeks*: *281*	*48 weeks*: *8 (7*.*9%); 96 weeks*: *19 (18*.*8%)*	*96 weeks VF*: *8/99 (8*.*1%)*	*3/8; IN N155/H and G163/R (1); IN N155/H*, *G163/R (1); IN N155H*, *T97A*, *D232N*, *Pr*. *M46I*, *V32I*, *I47V (1); IN G140/S*, *Q148/H (1)1/5*: *RT M184V (1)*
		*LPV/r + TDF/FTC (n = 105)*	*HIV-1 RNA <40 copies/mL*: *48 weeks*: *84*.*8% (ITT); 96 weeks*: *68*.*6% (ITT); 85*.*2% (OB)*	*48 weeks*: *245; 96 weeks*: *296*	*48 weeks*: *11 (10*.*5%); 96 weeks*: *15 (14*.*3%)*	*96 weeks VF*: *5/104 (4*.*8%)*	
**PI/r + MVC**	A4001078 [26,28,30]	ATV/r + MVC (n = 60)	48 weeks: 74.6% (ITT); 96 weeks: 67.8% (ITT)	48 weeks: 215; 96 weeks: 269	48 weeks: 7 (11.7%)	48 weeks TF: 2/59 (3.4%); 96 weeks TF: 3/59 (5.1%)	None detected
		ATV/r + TDF/FTC (n = 61)	48 weeks: 83.6% (ITT); 96 weeks: 82.0% (ITT)	48 weeks: 226; 96 weeks: 305	48 weeks: 7 (11.5%)	48 weeks TF: 2/61 (3.3%); 96 weeks TF: 2/61 (3.3%)	None detected
	MIDAS [31]	DRV/r + MVC (N = 24)	24 weeks: 87.5%; 48 weeks: 83.3%	247	Not reported	24 weeks VF: 3/24 (12.5%); 48 weeks VF: 4/24 (16.7%)	Not reported
	MODERN [32]	DRV/r + TDF/FTC (n = 406)	86.8%	48 weeks: 194	48 weeks: 50/401 (12.5%)	48 weeks TF: 13/401 (3.2%)	Not reported
		DRV/r + MVC (n = 406)	77.3%	48 weeks: 195	48 weeks: 73/396 (18.4%)	48 weeks TF: 40/396 (10.1%)	Not reported
	VEMAN [27,29]	LPV/r + MVC (n = 26)	100% (PP: 26/26)	286	None reported	None (PP)	None reported
		LPV/r + TDF/FTC (n = 24)	96% (PP: 22/24)	199	None reported	None reported (PP)	None reported
**PI/r + 3TC**	LOREDA [33]	LPV/r + 3TC (N = 39)	HIV-1 RNA<48 copies/mL: 66.7% (ITT)	250	12 (31%)	Discontinuations due to VF 44 weeks: 5/39 (12.8%)	No PI mutations; M184V in 3/3 tested
	*GARDEL [34]*	*LPV/r + 3TC (n = 217)*	*88*.*3%*	*227*	*16 (4*.*5%)*	*48 weeks VF*: *10/214 (4*.*7%)*	*No primary PI mutations*, *M184V in 2/5 tested*
		*LPV/r + 3TC or FTC + third NRTI (n = 209)*	*83*.*7%*	*217*	*27 (13*.*4%)*	*48 weeks VF*: *12/202 (5*.*9%)*	*0/7 tested*
**PI/r + NNRTI**	BMS-121 [35]	ATV/r (300/100 mg) + EFV (n = 32)	63%(ITT)	271	5/32 (16%)	Not reported	Not reported
		ATV/r (400/100 mg) + EFV (n = 33)	61% (ITT)	250	7/33 (21%)	Not reported	Not reported
	ACTG 5142 [36,37]	EFV + LPV/r (n = 250)	96 weeks: 83% (PP)	96 weeks: 273	164 patients (22%)	TF during median 112 weeks follow-up: 108/250 (43%); VF: 73/250 (29%)	16%; NNRTI- associated: 37, K103N (31); PI-associated: 45, major (2); NRTI-associated: 6, M184V (1)
		LPV/r + 2 NRTIs (n = 253)	96 weeks: 77% (PP)	96 weeks: 287		TF during median 112 weeks follow-up: 127/253 (50%); VF: 94/253 (37%)	6%; PI-associated: 61, major (0); NRTI-associated: 15, M184V (13); NNRTI-associated: 2
		EFV + 2 NRTIs (n = 250)	96 weeks: 89% (PP)	96 weeks: 230		TF during median 112 weeks follow-up: 95/250 (38%); VF: 60/250 (24%)	9%; NNRTI-associated: 20, K103N (11); NRTI-associated: 14, M184V (8), K65R (3); PI-associated: 39, major (0)
	MEDICLAS [38]	LPV/r + NVP (n = 26)	24 months: 81%	24 months: 308	7	Not reported	Not reported
		LPVr + ZDV/3TC (n = 22)	24 months: 77%	24 months: 280	9	Not reported	Not reported
	CTN 177 [39]	LPV/r + NVP (n = 26)	42%	225	AEs leading to discontinuation: 9/26	Not reported	Not reported
		NVP + ZDV/3TC (n = 26)	50%	134	AEs leading to discontinuation: 2/26	Not reported	Not reported
		LPV/r + ZDV/3TC (n = 25)	68%	190	AEs leading to discontinuation: 02/25	Not reported	Not reported

3TC, lamivudine; AE, adverse event; ARV, antiretroviral; ATV, atazanavir; ATV/r, atazanavir/ritonavir; DRV/r, darunavir/ritonavir; EFV, efavirenz; ITT, intent-to-treat; LPV/r, lopinavir/ritonavir; MVC, maraviroc; NNRTI, non-nucleoside reverse transcriptase inhibitor; NRTI, nucleoside reverse transcriptase inhibitor; NVP, nevirapine, OB = observed; PI/r, ritonavir-boosted protease inhibitor; PP, per-protocol; r, ritonavir; RAL, raltegravir; TDF/FTC, tenofovir/emtricitabine; TF, treatment failure; VF, virologic failure; ZDV, zidovudine.

^1^Rows with italicized text indicate randomised and sufficiently powered dual therapy studies that showed comparable outcomes with the standard therapy.

^2^VF/TF as defined per individual study protocol; proportion calculated using modified ITT population (patients receiving at least 1 dose of study drug), where available.

**Table 3:** Results of key secondary endpoints from identified trials among ARV-naive patients.

Regimen	Study Name	Treatment Arm	Adverse Events	Lipids	Renal	Bone	Lipoatrophy	Other Notes
**PI/r + RAL**	SPARTAN [16]	ATV + RAL (n = 63, 45 evaluable at week 48)	Grade 2–4 treatment-related AE hyperbilirubinemia, 17.5%; grade 4 hyperbilirubinemia, 20.6%	HDL ↑ by 24%; Total cholesterol ↑ by 7% at week 24	Not reported	Not reported	Not reported	Based on the higher rates of hyperbilirubinemia and the development of resistance to RAL, the regimen was not considered optimal to support further clinical development and therefore the trial was terminated early.
		ATV/r + TDF/FTC (n = 30, 25 evaluable at week 48)	Grade 2–4 treatment-related AE hyperbilirubinemia, 10.0%; Grade 4 hyperbilirubinemia, 0%	TG ↑ by 32%; total cholesterol ↑ by 10% at week 24	Not reported	Not reported	Not reported	
	RADAR [17,18]	DRV/r + RAL (n = 40)	3 severe AEs; none related to treatment	There were no statistically significant differences between groups in changes from baseline for total cholesterol, TG, total cholesterol/HDL ratio or serum creatinine at week 24		Patients in the TDF-containing arm had significantly more bone loss; markers of bone formation and destruction indicate a higher bone turnover	Not reported	
		DRV/r + TDF/FTC (n = 40)	1 severe AE; not related to treatment					
	ACTG A5262 [19]	DRV/r + RAL (N = 112)	Grade 3 or higher clinical or laboratory AEs were reported at least once by 19% of patients	Statistically significant median increases in: HDL: 9 mg/dL; LDL: 17 mg/dL; total cholesterol: 30 mg/dL; TG: 23 mg/dL	Not reported	Not reported	Not reported	Death occurred in one patient from cryptosporidiosis
	NEAT001/ ANRS143 [20]	DRV/r + RAL (n = 401)	Incidence rates of AEs (/100-PY) were similar between arms (TDF/FTC vs RAL): SAE, 8.3 vs 10.2; grade 3 or 4, 7.4 vs 9.6Statistically significant increases in RAL vs TDF/FTC arms at 96 weeks (mmol/L): TC– 0.9 vs 0.5 LDL– 0.5 vs 0.4	HDL– 0.2 vs 0.1 TC:HDL ratio did not change in either arm	eGFR (mL/min) changes at 96 weeks, RAL: +0.9; TDF/FTC: –3.8 (*P* = 0.02); mean changes in CrCl from BL to Wk 96 were +1.3 and -4.1 mL/min (*P* = 0.0004)	Not reported	Not reported	At 96 weeks, % of pts with grade 3/4 CK elevations, RAL:– 6.2; TDF/FTC:– 5.0 (*P* value not reported); AST/ALT elevations, RAL:– 3.0; TDF/FTC:– 1.0 (*P* value not reported)
		DRV/r + TDF/FTC (n = 404)				Not reported	Not reported	
	CCTG 589 [21]	LPV/r + RAL (n = 26)	No serious AEs; no difference in time to grade 2–4 AEs	Median cholesterol and TG at week 48 were non-significantly higher with LPV/r + RAL than EFV/TDF/FTC	Not reported	Not reported	Not reported	
		EFV/TDF/FTC (n = 25)	2 serious AEs (including 1 possibly related suicide ideation					
	PROGRESS [22–25]	LPV/r + RAL (n = 101)	AE profile and laboratory abnormalities were generally similar	No statistically significant differences between arms in mean change of lipid levels or lipid ratios (LDL:HDL and TC:HDL)	96 weeks: eGFR, −1.43 mL/min	DEXA 96 weeks (n = 78) Mean % change from BL for spine BMD, 1.34% and total BMD, 0.68%	Both regimens restored peripheral body fat; LPV/r + RAL increases in upper and lower extremity fat were statistically significantly higher vs LPV/r +TDF/FTC	
		LPV/r + TDF/FTC (n = 105)			96 weeks: eGFR, −7.33 mL/min	DEXA 96 weeks (n = 82) Mean % change from BL for spine BMD: -4.61%; total BMD: -2.48%; significant difference between arms		
**PI/r + MVC**	A4001078 [26,28,30]	ATV/r + MVC (n = 60)	Grade 3 or 4 AE, 48.3% (36.7% hyperbilirubinemia)	Not reported	48 weeks: CrCl stable; 96 weeks: CrCl: –5.5 mL/min	Formation markers were significantly different between arms	Not reported	10 patients switched from ATV/r to another PI (7 DRV/r; 3 LPV/r) for tolerability or unconjugated hyperbilirubinemia
		ATV/r + TDF/FTC (n = 61)	Grade 3 or 4 AE, 29.5% (19.7% hyperbilirubinemia)	Not reported	48 weeks: CrCl decreased; 96 weeks: CrCl: –18 mL/min		Not reported	
	MIDAS [31]	DRV/r + MVC (N = 24)	Not reported	LDL cholesterol increased to grade 3 in one patient	Not reported	Not reported	Not reported	
	MODERN [32]	DRV/r + TDF/FTC (n = 406)	Grade 3 or 4 AEs similar between groups	Not reported	Not reported	Not reported	Not reported	
		DRV/r + MVC (n = 406)	Not reported	Not reported	Not reported	Not reported	Not reported	
	VEMAN [27,29]	LPV/r + MVC (n = 28, 19 evaluable)	No grade 3 or grade 4 AEs	Cholesterol (total, HDL, LDL), and TG stable; no significant difference between arms	Not reported	Not reported	Not reported	Glucose and insulin stable; no significant difference between arms
		LPV/r + TDF/FTC (n = 27, 19 evaluable)	Diarrhoea led to 3 treatment interruptions					
**PI/r + 3TC**	LOREDA [33]	LPV/r + 3TC (N = 39)	Not reported	Not reported	Not reported	Not reported	Not reported	
	GARDEL [34]	LPV/r + 3TC (n = 217)	65 grade 2 or 3 AEs, 43 patients with grade 2–3 AEs	11% with grade 2–3 AEs	Not reported	Not reported	Not reported	
		LPV/r + 3TC or FTC + third NRTI (n = 209)	88 grade 2 or 3 AEs, 48 patients with grade 2–3 AEs	8% with grade 2–3 AEs	Not reported	Not reported	Not reported	
**PI/r + NNRTI**	BMS-121 [35]	ATV/r (300/100) + EFV (n = 32)	Grade 2–4 AEs = 26%	Mean change from BL (%) in total cholesterol, LDL, HDL and TG = 29, 11, 54, and 48, respectively.	Increase in grade 3–4 total bilirubin, ALT, and AST by 13%, 10%, and 7%.	Not reported	Not reported	
		ATV/r (400/100) + EFV (n = 33)	Grade 2–4 AEs = 30%	Mean change from BL (%) in total cholesterol, LDL, HDL, and TG = 32, 13, 45, and 63, respectively.	Increase in grade 3–4 total bilirubin, ALT, and AST by 40%, 7%, and 3%	Not reported	Not reported	
	ACTG 5142 [36,37]	EFV + LPV/r (n = 250)	Grade 3 or 4 new clinical event = 43 (17%); Grade 3 or 4 laboratory abnormality = 107 (43%)	LDL >190 mg/dL = 14 (6%) TG >750 mg/dL = 34 (14%)	Creatinine kinase >5 times ULN = 14 (6%)	Not reported	Clinical lipoatrophy = 0; mean limb fat increases: 1.1 kg	
		LPV/r + 2 NRTIs (n = 253)	Grade 3 or 4 new clinical event = 46 (18%); Grade 3 or 4 laboratory abnormality = 80 (32%)	LDL >190 mg/dL = 2 (1%) TG >750 mg/dL = 16 (6%)	Creatine kinase >5 times ULN = 8 (3%)	Not reported	Clinical lipoatrophy = 3 (1%); mean limb fat increases: 0.7 kg	
		EFV + 2 NRTIs (n = 250)	Grade 3 or 4 new clinical event = 42 (17%); Grade 3 or 4 laboratory abnormality = 72 (29%)	LDL >190 mg/dL = 7 (3%) TG >750 mg/dL = 6 (2%)	Creatine kinase >5 times ULN = 8 (3%)	Not reported	Clinical lipoatrophy = 8 (3%) mean limb fat increases: 0.05 kg	
	MEDICLAS [38]	LPV/r + NVP (n = 26)	Grade 3 or 4 AEs, 54.2%	Total cholesterol and HDL increased by 36.5% and 38/8%	Not reported	Not reported	At 24 months, total fat increased to 15643 g.	
		LPV/r + ZDV/3TC (n = 22)	Grade 3 or 4 AEs, 45.8%	Total cholesterol and HDL increased by 23.2% and 32.8%	Not reported	Not reported	At 24 months, total fat increased to 14254 g	
	CTN 177 [39]	LPV/r + NVP (n = 26)	Grade 3 or 4 AEs, 34.6%	Median changes from BL to week 48 in TC, HDL, and TG of +1.8, +0.6, and +0.4 mmol/L, respectively	Not reported	Not reported	Not reported	
		NVP + ZDV/3TC (n = 25)	Grade 3 or 4 AEs, 7.4%	Median changes from BL to week 48 in TC, HDL, and TG of +0.8, +0.4, and –0.1 mmol/L, respectively	Not reported	Not reported	Not reported	
		LPV/r + ZDV/3TC (n = 25)	Grade 3 or 4 AEs, 8.33%	Median changes from BL to week 48 in TC, HDL, and TG of +1.3, +0.2, and +0.9 mmol/L, respectively	Not reported	Not reported	Not reported	

3TC, lamivudine; AE, adverse event; ALT, alanine aminotransferase; ARV, antiretroviral; AST, aspartate aminotransferase; ATV, atazanavir; ATV/r, atazanavir/ritonavir; BL, baseline; BMD, bone mineral density; DRV/r, darunavir/ritonavir; eGFR, estimated glomerular filtration rate; EFV, efavirenz; HDL, high-density lipoprotein; ITT, intent-to-treat; LDL, low-density lipoprotein; LPV/r, lopinavir/ritonavir; MVC, maraviroc; NNRTI, non-nucleoside reverse transcriptase inhibitor; NRTI, nucleoside reverse transcriptase inhibitor; NVP, nevirapine, PI/r, ritonavir-boosted protease inhibitor; PP, per-protocol; PY, person-years; r, ritonavir; RAL, raltegravir; SAE, serious adverse event; TDF/FTC, tenofovir/emtricitabine; TG, triglycerides; ZDV, zidovudine.

### PIs in Combination With Raltegravir

#### Atazanavir + raltegravir therapy

The SPARTAN study [16] (a noncomparative pilot study; N = 93) compared atazanavir (ATV) + raltegravir (RAL) with ATV/r + tenofovir/emtricitabine (TDF/FTC) and was terminated early. Although the efficacy of ATV + RAL was similar to ATV/r + TDF/FTC, combination therapy with ATV + RAL was associated with a high rate of severe hyperbilirubinaemia. Despite a high dose of ATV (300 mg twice daily [BID]), the development of RAL resistance was seen in regimen failures.

#### Darunavir/r + RAL therapy

Results from studies evaluating darunavir (DRV)/r plus RAL are mixed. The RADAR study [17,18] (N = 80) provided some initial evidence for the effectiveness of DRV/r + RAL therapy; however, the 48-week results did not confirm the noninferiority of the DRV/r + RAL arm because of a higher level of discontinuations compared with the DRV/r + TDF/FTC arm. Likewise, in ACTG A5262 [19], DRV/r + RAL therapy demonstrated poorer than expected results in this single-arm phase 2 study. Among 112 patients, HIV-RNA levels were not suppressed in 11 patients and 6 and 11 patients rebounded by weeks 24 and 48, respectively, resulting in a 26% failure rate overall [19]. Although high baseline viral loads and poor adherence could have contributed to the poor results, the lack of a comparator arm limits the assessment of these data.

The NEAT 001 study [20], a large (N = 805) noninferiority randomised open-label study comparing the efficacy and safety of DRV/r in combination with either TDF/FTC or RAL, utilized time to treatment failure (virologic or clinical) as the primary endpoint. Per Kaplan-Meier methodology, therapy failure occurred in an estimated 17.4% of patients in the RAL arm and 13.7% in the TDF/FTC arm after 96 weeks (adjusted difference, 3.7% [95% CI, −1.1 to 8.6]), falling within a pre-specified noninferiority margin of 9%. In subgroup analyses, patients with CD4 counts <200 cells/μL had substantially higher rate of treatment failures using RAL therapy compared with TDF/FTC therapy. The reasons for the less than optimal treatment response in the non-NRTI arm in this subset of patients are being studied further. Although the number of virologic failures was low, 5 patients in the RAL arm developed integrase resistance, whereas no patients in the TDF/FTC arm had with PI resistance.

#### LPV/r +RAL Therapy

In the CCTG 589 pilot trial [21] (N = 51) comparing therapy with LPV/r + RAL with efavirenz (EFV) + TDF/FTC, a high discontinuation rate (19.2%) decreased the proportion of patients who achieved viral suppression with LPV/r + RAL (69%; ITT analysis); however, by observed analysis, 86% of patients achieved HIV-1 RNA levels <50 copies/mL at 48 weeks. In the PROGRESS trial [22–25], the noninferiority of LPV/r + RAL (n = 101) to LPV/r + TDF/FTC (n = 105) was demonstrated at 48 weeks (using a 20% noninferiority margin), with 83.2% and 84.8% of patients achieving HIV-1 RNA levels <40 copies/mL, respectively (ITT analysis; difference, −1.6%; 95% CI, −12.0% to 8.8%). At week 96, 66.3% and 68.6% of patients had viral load suppression (<50 copies/mL; ITT analysis). LPV/r + RAL was generally well-tolerated [22–25].

### PIs in Combination With Maraviroc

Four studies assessed a PI/r in combination with maraviroc (MVC). The VEMAN (N = 50) and A4001078 (N = 121) studies demonstrated virologic suppression with LPV/r + MVC and ATV/r + MVC, respectively, in ARV-naive patients [26–30]. In the A4001078 study, the grade 3 and 4 elevations in bilirubin levels in patients treated with ATV/r + MVC (36.7%) versus ATV/r + TDF/FTC (19.7%) were of concern. Ten patients in this study switched from ATV/r to DRV/r or LPV/r due to toxicity issues [26,30]. Whether the ATV/r + MVC regimen is associated with improvement with other toxicity issues will require further examination of between-group differences in bone and immune activation markers [28]. In the MIDAS study (DRV/r + MVC; N = 24), the rate of VF was high, especially in patients with higher baseline viral loads; the virus was not suppressed at 48 weeks in 16.7% patients (4/24), despite reported perfect adherence to therapy [31].

A fourth study, A4001095 [32] (MODERN; N = 812), was designed to assess therapy with DRV/r + TDF/FTC and DRV/r + once-daily MVC, and utilized 150 mg MVC with DRV/r 800/100 mg once daily. This study was terminated early because of inferior efficacy in the MVC arm [32]. A switch to this combination in patients with virologic suppression remains under study in the MARCH trial, which has been fully recruited; however, results have not yet been reported.

### PIs in Combination With Lamivudine

Two studies examined a PI/r in combination with lamivudine (3TC). In a single-arm pilot study (LOREDA, N = 39) [33], the LPV/r + 3TC combination demonstrated moderate virologic efficacy (66.7% of patients with viral load <48 copies/mL, ITT; 81.2%, as treated); however, VF was high (13%) [33]. The GARDEL study (N = 426) was a randomised, controlled, and powered study that compared LPV/r + 3TC with LPV/r + 2 NRTIs [34]. LPV/r + 3TC was noninferior to standard triple therapy at 48 weeks regardless of baseline viral loads (<50 copies/mL: dual therapy, 88.3%; triple therapy, 83.7%; *P* = 0.171). There were fewer discontinuations in the LPV/r + 3TC arm largely because of safety and toxicity reasons. VF occurred at low levels in both treatment arms and did not result in any PI resistance in either arm. The M184V mutation was identified in 2 of 5 patients in the LPV/r + 3TC arm. Because the second NRTI in the triple therapy arm was most commonly zidovudine (ZDV), the generalizability of these results to all NRTIs may be limited. However, when the comparison was limited to non–ZDV-containing regimens, noninferiority was confirmed.

### PIs in Combination With NNRTIs

Four studies evaluating a PI/r (LPV/r or ATV/r) in combination with an NNRTI (EFV) were identified. In BMS-121 [35] (N = 65), which compared ATV/r (300/100 mg) + EFV with ATV/r (400/100 mg) + EFV, rates of virologic suppression were similar with either treatment combination. In ACTG 5142 [36,37] (N = 753), time to VF was similar in the LPV/r + EFV arm when compared with the triple therapy arms; however, resistance (any mutation [excluding minor protease mutations] and NNRTI-associated mutations) and grades 3 and 4 laboratory events were more common with LPV/r + EFV. In the MEDICLAS study [38] (N = 48), patients receiving LPV/r + nevirapine (NVP) or LPV/r + ZDV/3TC had similar rates of virologic suppression (80% and 77%, respectively). In the CTN 177 study [39] (N = 77), rates of virologic suppression were lower with LPV/r + NVP versus NVP + ZDV/3TC or LPV/r + ZDV/3TC. Discontinuations related to AEs (rash and elevated transaminases) were more frequent in the LPV/r + NVP arm compared with the other treatment arms [39].

### Trials of ARV-Experienced, Virologically Suppressed Patients

Another approach to an ARV-sparing strategy is to suppress patient HIV-RNA levels using standard triple ARV regimens and then switch or simplify to a dual therapy regimen. Thirteen trials were identified that examined novel dual therapy regimens in ARV-experienced, virologically suppressed patients. Trial designs and key findings from these studies are summarised in Tables 4, 5 and 6. Virologic efficacy endpoints are summarised in Fig 2B.

**Table 4:** Study designs for identified trials in ARV-experienced, virologically suppressed patients.

Regimen	Study Name	Duration	Type	Suppression Criteria	Treatment Arm	Dose	Primary Endpoint
**PI/r + RAL**	BATAR [40]	48 weeks	Open-label, exploratory, pilot trial	HIV viral load <50 copies/mL	ATV/r + RAL (arm 1; n = 15)	300/100 mg QD + 400 mg BID	Maintenance of virologic suppression (<40 c/mL); all but two patients maintained virologic suppression; both virologic failures (>200 c/mL on two consecutive tests) were on arm 2 (ATV + RAL, both BID)
					ATV + RAL (arm 2; n = 14)	300 mg BID + 400 mg BID	
					ATV/r + TDF/FTC (ctrl; n = 14)	300/100 mg QD + 300/200 mg QD	
	Ruane [41]	48 weeks, switch to 96 weeks	Prospective, single-centre, single-arm switch study, with extension	HIV viral load <48 copies/mL	ATV+ RAL (N = 30)	400 mg QD + 400 mg BID	VL <48 copies/mL by week 48
	SPARE (ongoing) [42]	96 weeks	Multicenter, phase 3b, randomised, open-label, parallel group study	HIV-1 RNA viral load of <50 copies/mL over a period of >15 weeks	DRV/r + RAL (n = 29)	800/100 mg QD + 800 mg QD	The proportion of patients with >10% improvement in eGFR at 48 weeks from baseline
					LPV/r + TDF/FTC (n = 30)	800/200 mg per day + 300/200 mg QD	
	Calza 2013 [43]	12 months	Prospective, observational	HIV viral load <50 copies/mL	DRV/r + RAL (N = 71)	800/100 mg QD + 400 mg BID	Virologic efficacy and safety
	KITE [44]	48 weeks	Randomised, prospective, open-label pilot study	<50 copies/mL for >6 months) previously on standard HAART (2 NRTIs + PI or NNRTI	LPV/r + RAL (n = 40)	400/100 mg BID + 400 mg BID	HIV-1 RNA <50 copies/mL at week 48 (ITT): 91.7% vs 88.2%
					HAART (n = 20)	NA	
**PI/r + 3TC**	ATLAS [46,47]	48 weeks extended to 96 weeks	Single-arm, prospective, pilot study	<50 copies/mL for >3 months) previously on ATV/r-based regimen (39 TDF; 1 abacavir)	ATV/r + 3TC (n = 40)	300/100 mg QD + 300 mg QD	Stopping rule set at 5 VF defined as HIV-RNA>50 c/mL
	SALT [48]	96 weeks	Randomised, open-label	HIV viral load <50 copies/mL	ATV/r + 3TC (n = 64)	ATV/r (300 mg/100 mg QD) + 3TC (300 mg QD)	VL <50 c/mL: 87.5% (dual) vs 92.5% (triple) (difference –5%; 99.95% CI, −26.3% to 15.5%)
					ATV/r + 2 NRTIs (n = 67)		
**PI/r + NNRTI**	A5116 [50]	72 weeks	Multicenter, randomised, open-label study	<200 copies/mL for ≥18 months) previously on stable 3 or 4 PI- or NNRTI-based regimen	EFV + LPV/r (n = 118)	600 mg QD + 533/133 mg BID	Time to confirmed VF (2 consecutive HIV-1 RNA >200 copies/mL)
					EFV + 2 NRTIs (n = 118)	600 mg QD + as appropriate	
	NEKA [51]	48 weeks	Randomised, open-label pilot study	<80 copies/mL previously on the same PI- or NNRTI-based regimen for >9 months	LPV/r + NVP (n = 16)	400/100 mg BID + 2 NRTIs	HIV-1 RNA <80 copies/mL at week 48 (ITT): 87.5% vs 100%
					LPV/r + 2 NRTIs (n = 15)	400/100 mg BID + As appropriate	
**RAL + NNRTI**	Reliquet 2014 [52]	36 months	Retrospective	<50 copies/mL for more than 6 months on an NVP-containing regimen	RAL + NVP (N = 39)	400 mg BID + 400 mg QD	Not reported
	Calin 2013 [53]	52 weeks	Observational, single centre	<50 copies/mL	RAL + ETR (N = 91)	400 mg BID + 200 mg BID	Continued virologic suppression (<50 copies/mL)
**RAL + MVC**	ROCnRAL ANRS157 [54]	Median time 19.4 weeks (stopped early)	Non-comparative, phase 2 pilot study	HIV-RNA <200 copies/mL for last 24 months and <50 copies/mL for ≥12 months	RAL + MVC (N = 44)	400 mg BID + 300 mg BID	Virologic failure defined as 2 plasma viral load measurements >50 copies/mL
	No Nuc No Boost [55]	48 weeks	Open-label, single-arm, phase 2	HIV-RNA <50 copies/mL at week 20 and 22	RAL + MVC (N = 10)	400 mg BID + 300 mg BID	HIV-RNA <50 copies/mL at week 48

3TC, lamivudine; ARV, antiretroviral; ATV/r, atazanavir/ritonavir; BID, twice a day; DRV/r, darunavir/ritonavir; EFV, efavirenz; eGFR, estimated glomerular filtration rate; ETR = etravirine; HAART, highly active antiretroviral therapy; ITT, intent-to-treat; LPV/r, lopinavir/ritonavir; MVC, maraviroc; NNRTI, non-nucleoside reverse transcriptase inhibitor; NRTI, nucleoside reverse transcriptase inhibitor; NVP, nevirapine; PI/r, ritonavir-boosted protease inhibitor; PP, per-protocol; QD, once a day; RAL, raltegravir; TDF, tenofovir; TDF/FTC, tenofovir/emtricitabine; TF, treatment failure; VF, virologic failure; VL, viral load.

**Table 5:** Results of key efficacy endpoints from identified trials in ARV-experienced virologically suppressed patients.

Regimen	Study Name	Treatment Arm	HIV-1 RNA <50 Copies/mL at Week 48 (Unless Specified)	Mean/Median CD4 Increase (cells/mm^3^) at Week 48 (Unless Specified)	Discontinuations, n	Treatment Failures/ Virologic Failures^1^, n (%)	Mutations
**PI/r + RAL**	BATAR [40]	ATV/r + RAL (n = 15)	48 weeks: 100% (15/15)	Overall CD4 counts were 534/mm^3^ at BL and 555/mm^3^ at week 48. There was a significant CD4 cell count difference favouring ATV/r+ TDF/FTC (52/mm^3^) vs arm 2 (−14/mm^3^); *P* = 0.03	Not reported	48 weeks VF: 0/15 (0%)	Not reported
		ATV + RAL (n = 14)	48 weeks: 85.7% (12/14)			48 weeks VF: 2/14 (14.3%)	None detected
		ATV/r + TDF/FTC (n = 14)	48 weeks: 100% (14/14)			48 weeks VF: 0/14 (0%)	Not reported
	Ruane [41]	ATV + RAL (N = 30)	23/30 patients remain on protocol (median, 72 weeks; range, 36–96) and all have HIV VL <48 copies/mL	The median (range) increase (LOCF) in the absolute CD4 count from BL to week 48 was 64 (53–100) cells/mm^3^	2	VF: 4/30 (13.3%)	Not reported
	SPARE (ongoing) [42]	DRV/r + RAL (n = 29)	24 weeks: 96.2% (PP), 89.3% (ITT); 48 weeks: 100% (PP), 85.7% (ITT)	Not reported	4	None reported	Not reported
		LPV/r + TDF/FTC (n = 30)	24 weeks: 96.7% (PP and ITT); 48 weeks: 100% (PP), 96.7% (ITT)	Not reported	1	None reported	Not reported
	Calza 2013 [43]	DRV/r + RAL (N = 71)	12 months: 94% (ITT)	123	4	12 months VF: 1/71 (1.4%)	None reported
	KITE [44]	LPV/r + RAL (n = 40)	91.7% (ITT)	519	5	48 weeks TF: 4/39 (10.3%);1/39 (2.6%) due to VF	None reported
		HAART (n = 20)	88.2% (ITT)	576	1	48 weeks TF: 2/20 (10.0%); both due to VF)	None reported
**PI/r + 3TC**	ATLAS [45–47]	ATV/r + 3TC (N = 40)	Week 48: 90% (ITT); Week 96: 85% (ITT)	33	Not reported	48 weeks TF: 5/38 (13.2%); VF: 2/38 (5.3%); 96 weeks VF: 1/40 (2.5%)	0/2
	SALT [48]	ATV/r + 3TC (n = 64)	Week 24: 87.5%	57	8	Week 24 VF: 0/64 (0%)	NA
		ATV/r + 2 NRTIs (n = 67)	Week 24: 92.5%	−27	5	Week 24 VF: 0/67 (0%)	NA
**PI/r + NNRTI**	A5116 [50]	EFV + LPV/r (n = 118)	Not reported	48 weeks: 40.4; 96 weeks: 67.8	20	TF during median 110 weeks follow-up: 34/115 (29.6%); VF: 14/115 (12.2%)	In reverse transcriptase, M184V (1), K103N (5), V106A/M (2), Y188H (1), G190A (2), V108I (1). In protease, L33V (2) and F53L (1).
		EFV + 2 NRTIs (n = 118)	Not reported	48 weeks: 17.4; 96 weeks: 43.6	14	TF during median 110 weeks follow-up: 13/117 (11.1%); VF: 7/117 (6.0%)	In reverse transcriptase, M184V/I (5), K103N (5), V106M (1), M230L (1), P225H (1). In protease, L33V (1)
	NEKA [51]	LPV/r + NVP (n = 16)	HIV-1 RNA <80 copies/mL: 87.5% (NVP group) and 100% NRTI group	300	2	Not reported	Not reported
		LPV/r + 2 NRTIs (n = 15)		155	0		
**RAL + NNRTI**	Reliquet 2014 [52]	RAL + NVP (N = 39)	6 months 87.2% (ITT); 97.1% (PP); 12 months 82.1% (ITT); 94.1% (PP)	Not reported	6	VF during median 27 months follow-up: 1/39 (2.6%)	In 1 patient at VF, G190A, G140G/S, Q148H
	Calin 2013 [53]	RAL + ETR (N = 91)	6 months: 98.2% (PP); 12 months: 92.3% (PP)	Not reported	18	VF during median 11.5 months follow-up: 5/91 (5.5%)	In 3/5 cases, VF was followed by acquired RAL (N155H, Q148H) and ETR (Y181V) mutations
**RAL + MVC**	ROCnRAL ANRS157 [54]	RAL + MVC (N = 44)	For virologic reasons, study was discontinued early. At end, all but 3 were ART-controlled	No change	4	24 weeks TF: 7/44 (15.9%); VF: 5/44 (11.4%)	Resistance mutations emerged in 3/5 VF patients: Y143C, N155H, F121Y
	No Nuc No Boost [55]	RAL + MVC (N = 10)	100%	Approximately 750	0	48 weeks VF: 0/10 (0.0%)	Not reported

3TC, lamivudine; ART, antiretroviral therapy; ARV, antiretroviral; ATV/r, atazanavir/ritonavir; BL, baseline; DRV/r, darunavir/ritonavir; EFV, efavirenz; ETR = etravirine; HAART, highly active antiretroviral therapy; ITT, intent-to-treat; LOCF, last observation carried forward; LPV/r, lopinavir/ritonavir; MVC, maraviroc; NA, not available; NNRTI, non-nucleoside reverse transcriptase inhibitor; NRTI, nucleoside reverse transcriptase inhibitor; NVP, nevirapine; PI/r, ritonavir-boosted protease inhibitor; PP, per protocol; r, ritonavir; RAL, raltegravir; TDF, tenofovir; TDF/FTC, tenofovir/emtricitabine; TF, treatment failure; VF, virologic failure; VL, viral load.

^1^VF/TF as defined per individual study protocol; proportion calculated using modified ITT population (patients receiving at least 1 dose of study drug), when available.

**Table 6:** Results of key secondary endpoints from identified trials among ARV-experienced HIV-suppressed patients.

Regimen	Study Name	Treatment Arm	Adverse Events	Lipids	Renal	Bone	Lipoatrophy
**PI/r + RAL**	BATAR [40]	ATV/r + RAL (arm 1; n = 15)	Neurologic, n = 7; musculoskeletal, n = 3	No significant difference between groups	Not reported	Not reported	Not reported
		ATV + RAL (arm 2; n = 14)	Neurologic, n = 6; musculoskeletal, n = 7				
		ATV/r + TDF/FTC (control, n = 14)	Neurologic, n = 1; musculoskeletal, n = 1				
	Ruane [41]	ATV + RAL (N = 30)	AEs were generally mild to moderate in severity. No grade 4 AEs were reported. Two grade 3 AEs were reported, nausea and weight loss, both of which were considered not related to study drug	Decreases in TC, TG, LDL, and HDL were observed	Not reported	Not reported	Not reported
	SPARE (ongoing) [42]	DRV/r + RAL (n = 29)	Grade 3 or 4 AEs at least one grade higher than baseline: rise in ALT (due to acute hepatitis B infection, n = 1) and elevated LDL cholesterol (n = 3)	Not reported	At week 48, 6/24 (25%) experienced >10% improvement in eGFR from baseline	Not reported	Not reported
		LPV/r + TDF/FTC (n = 30)	Grade 3 or 4 AEs at least one grade higher than baseline: elevated LDL cholesterol (n = 1), and hypophosphatemia (n = 3)	Not reported	At week 48, 3/28 (11%) experienced >10% improvement in eGFR from baseline	Not reported	Not reported
	Calza 2013 [43]	DRV/r + RAL (N = 71)	Gastrointestinal symptoms, n = 3; virologic failure (n = 1)	Mean TG −57 mg/dL (*P*<0.05) compared with baseline	Significant decrease in number of patients with proteinuria from 31% to 15% (*P*<0.05)	Not reported	Not reported
	KITE [44]	LPV/r + RAL (n = 40)	No serious AEs during the study; other than myalgia (25% in HAART vs 0), no differences between groups in AEs; trend towards more diarrhoea in LPV/r + RAL	Adjusted mean HDL levels and LDL levels were not statistically significantly different between groups. Total cholesterol and TG significantly lower in HAART arm at week 24	Adjusted creatinine clearance was not statistically significantly different between groups.	No significant differences between LPV/r + RAL (n = 37) and HAART (n = 18) in DEXA scans for BMD	No significant differences between LPV/r + RAL (n = 37) and HAART (n = 18) in DEXA scans for total body fat composition
		HAART (n = 20)					
**PI/r + 3TC**	ATLAS [45–47]	ATV/r + 3TC (N = 40)	6 severe AEs at 48 weeks: 4 renal colic, 1 hypertensive crisis, 1 brain haemorrhage	Significant changes at 48 weeks: TC, +15 mg/dL; HDL, +6 mg/dL; 96 weeks: TC, +19 mg/dL; HDL, +5 mg/dL	Significant changes at 48 weeks: eGFR CG: 6 mL/min; 96 weeks: MDRD: 15 mL/min	48 weeks: trend towards increase in L2–L4 BMD: 0.01 g/cm^2^; osteoporosis in 7% BL and 8% at 48 weeks; osteopenia in 40% and 38%, respectively; significant decreases in osteocalcin (−12 ng/mL) and alkaline phosphatase (−40 UI/L); no significant changes were observed in serum calcium, PTH, or vitamin D	At 48 weeks, significant increases in subcutaneous fat in cheek: +0.54 g and upper limb: +145 g, but not lower limb
	SALT [48]	ATV/r + 3TC (n = 64)	2 serious AEs, acute pyelonephritis, n = 1;traumatic bone fracture, n = 1	Not reported	Not reported	Not reported	Not reported
		ATV/r + 2 NNRTI (n = 67)	1 serious AE: toxicity due to drugs of abuse, n = 1	Not reported	Not reported	Not reported	Not reported
**PI/r + NNRTI**	A5116 [50,87]	EFV + LPV/r (n = 118)	No difference in time to grade 3 or 4 AEs. Trend towards greater rate of first grade 3 or 4 laboratory abnormality in EFV + LPV/r arm, largely due to TG	Cholesterol increased with LPV/r + EFV compared to minimal changes with EFV + NRTI. Non-HDL cholesterol increased with LPV/r + EFV and decreased with EFV+NRTI. HDL increased in both groups. Greater increases in TG with LPV/r + EFV	Not reported	48 weeks: no significant change for either group for lumbar or hip BMD or bone markers (osteocalcin and NTX)	Not reported
		EFV + 2 NRTIs (n = 118)					
	NEKA [51]	LPV/r + NVP (n = 16)	Proportion of patients with AEs was similar between arms. GI symptoms were most frequently reported	By week 48, TC, HDL, and TG increased by 14%, 11%, and 56%, respectively	Not reported	Not reported	Two patients had marked improvement in peripheral lipoatrophy
		LPV/r + 2 NRTIs (n = 15)		By week 48, TC, HDL, and TG increased by 13%, decreased bu11%, and increased by 18%, respectively	Not reported	Not reported	Two patients saw improvement in peripheral lipoatrophy
**RAL + NNRTI**	Reliquet 2014 [52]	RAL + NVP (N = 39)	4 discontinuations due to AEs: arthralgia, abdominal pain, weight gain, and neuropsychologic disorders	Lipid profile improved at 6 months for all parameters (*P*<0.05) except LDL. Median TC, –21 mg/dL, HDL, +3.74 mg/dL, and TG, –41 mg/dL)	SCr improved in all pts (–8.6 mmol/L) and in patients switched from TDF regimen (–9.75 mmol/L)	Not reported	Not reported
	Calin 2013 [53]	RAL + ETR (N = 91)	Possible causal relation with RAL/ETR therapy established in 5 patients (headache, dizziness, arthralgias, erectile dysfunction, and ETR hypersensitivity)	Not reported	Not reported	Not reported	Not reported
**RAL + MVC**	ROCnRAL ANRS157 [54]	RAL + MVC (N = 44)	2 discontinuations due to SAEs (grade 4 elevation in AST/ALT) and cutaneous rash and diarrhoea	Significant decrease in TG and TC; significant increase in LDL	Not reported	Significant increase in BMD	No significant difference in BMI, limb fat and trunk fat
	No Nuc No Boost [55]	RAK + MVC (N = 10)	1 grade 3 CPK elevation related to physical exercise	Not reported	Not reported	Not reported	Not reported

3TC, lamivudine; AE, adverse event; ALT, alanine aminotransferase; ARV, antiretroviral; AST, aspartate aminotransferase; ATV/r, atazanavir/ritonavir; AZT/3TC, zidovudine/lamivudine; BL, baseline; BMD, bone mineral density; BMI, body mass index; CG, Cockcroft-Gault; CPK, creatinine phosphokinase; CrCl, creatinine clearance; ddI/3TC, didanosine/lamivudine; DEXA, Dual-energy X-ray absorptiometry scan; DRV/r, darunavir/ritonavir; EFV, efavirenz; eGFR, estimated glomerular filtration rate; ETR = etravirine; GI, gastrointestinal; HAART, highly active antiretroviral therapy; HDL, high-density lipoprotein; LDL, low-density lipoprotein; LPV/r, lopinavir/ritonavir; MDRD, Modification of Diet in Renal Disease; MVC, maraviroc; NNRTI, non-nucleoside reverse transcriptase inhibitor; NRTI, nucleoside reverse transcriptase inhibitor; NTx, N-terminal telopeptide; NVP, nevirapine; PI/r, ritonavir-boosted protease inhibitor; PTH, parathyroid hormone; RAL, raltegravir; SAE, serious adverse event; SCr, serum creatinine; TDF/FTC, tenofovir/emtricitabine; TC, total cholesterol; TG, triglycerides.

### PIs in Combination With RAL

Five small studies assessed treatment simplification to PI/r + RAL-based regimens in virologically suppressed patients. In the BATAR study [40] (ATV/r + RAL, n = 15; ATV + RAL, n = 14; ATV/r + TDF/FTC, n = 14), 95% of patients (41/43) overall maintained viral suppression (≤200 copies/mL) at 48 weeks; 2 VFs occurred with ATV + RAL. In the Ruane study [41] (ATV + RAL [N = 30]), 23 patients who remained on protocol after a median of 72 weeks of therapy maintained virologic suppression (<48 copies/mL). In the ongoing SPARE study [42] (N = 59), which is evaluating DRV/r + RAL compared with LPV/r + TDF/FTC, all patients maintained virologic suppression (<50 copies/mL) at week 48. In the Calza 2013 study [43] (DRV/r + RAL; N = 71), 94% (67/71) of patients maintained viral suppression (<50 copies/mL) at 12 months. The KITE study [44] (N = 60), which assessed simplification to LPV/r + RAL from standard highly active antiretroviral therapy (HAART), demonstrated efficacy (virologic suppression <50 copies/mL) and safety comparable with continuing HAART over 48 weeks.

### PIs in Combination With 3TC

Two studies of a PI/r in combination with 3TC were identified. In the single-arm ATLAS trial [45–47] (N = 40), simplification to therapy with ATV/r + 3TC demonstrated maintenance of virologic efficacy (<50 copies/mL) and no grade 4 laboratory toxicities or treatment interruptions due to the development of new laboratory toxicities at 96 weeks. In the SALT study [48] (N = 131), 87.5% of virologically suppressed patients who received ATV/r + 3TC maintained virologic suppression (<50 copies/mL) compared with 92.5% of patients who received ATV/r + 2 NRTIs (95% CI, −26.3% to 15.5%) at 24 weeks. The OLE study is an open-label prospective study evaluating LPV/r + 3TC or FTC compared with LPV/r + 2 NRTIs, which is ongoing through 48 weeks; results have not yet been reported [49].

### PIs in Combination With NNRTIs

In A5116 [50] (N = 236), the combination of LPV/r + EFV was associated with increased toxicity-related discontinuations and a trend towards increased rates of VF (2 consecutive plasma HIV-1 RNA >200 copies/mL) compared with EFV + 2 NRTIs. In the NEKA study [51] (N = 31), in which virologically suppressed patients were switched to LPV/r + NVP or continued with LPV/r + 2 NRTIs, the proportion of patients who maintained virologic suppression were comparable, and LPV/r + NVP was generally well-tolerated over 48 weeks. Data regarding simplification of a 2-ARV regimen combining PI/r with rilpivirine, which could decrease pill burden and lessen toxicity, are not currently available.

### Integrase Inhibitors in Combination With NNRTIs

Reliquet 2014 [52] (N = 39) evaluated simplification of therapy to raltegravir (RAL) in combination with NVP. At 12 months following a switch of therapies from NVP + a non-RAL ARV, 82.1% of patients (ITT analysis) maintained virologic suppression (<50 copies/mL). All patients who reached month 24 (n = 22) or month 36 (n = 12) also maintained virologic suppression. Calin 2013 [53] was a study (N = 91) that evaluated simplification to RAL in combination with etravirine (ETR). At week 48, the discontinuation rate was 20%, including 3 patients who discontinued because of VF (2 consecutive plasma viral load >50 copies/mL).

### Integrase Inhibitors in Combination With MVC

Two studies examined simplification to RAL in combination with MVC from a suppressive ARV [54] or RAL + MVC + TDF/FTC [55]. The ROCnRAL ANRS 157 study [54] (N = 44) was discontinued because of a high rate (n = 5) of VF (2 consecutive plasma viral load >50 copies/mL) after a median duration of 20 weeks. In the No Nuc No Boost study [55] (N = 10), HIV-RNA levels remained <50 copies/mL in 9 of 10 patients following 44 weeks of treatment with RAL + MVC.

### Secondary Endpoint Findings From Trials of ARV-Naive and ARV-Experienced, Virologically Suppressed Patients

The main rationale for dual therapy is to optimize health status and quality of life without compromising control of HIV infection. Toxicities and comorbidities may interfere with patient quality of life and negatively affect adherence to ARV regimens and can lead to additional costs for diagnosis and management. Thus, an ideal regimen would provide potent virologic suppression while conferring a lower risk of long-term, ARV-related AEs and would minimally affect any comorbidities that may be present.

No study thus far has been able to examine the impact of dual therapy on these outcomes in the long-term. Key secondary endpoint findings from studies identified in this report are summarised in Tables 3 and 6.

### Renal Markers

Renal impairment, which is occasionally observed among individuals with HIV infection, may be related to chronic HIV infection, underlying comorbidities such as diabetes or hypertension, the use of medications, or a combination of factors [9,56–58]. Cohort studies have demonstrated that cumulative exposure to some ARVs, for example, TDF, is associated with increased rates of chronic kidney disease or decreases in the estimated glomerular filtration rate (eGFR) [9,56,57]. Sparing such agents may help preserve renal function.

Few studies included in this report reported renal function as an outcome. In the PROGRESS trial [24], a significantly greater decrease in eGFR from baseline was noted with LPV/r + TDF/FTC therapy than with LPV/r + RAL (−7.33 mL/min vs −1.43 mL/min, respectively; *P* = 0.035), potentially due to the presence of TDF. A similar result was observed in the NEAT 001 study [20], where a reduction in eGFR was significantly greater in the DRV/r + TDF/FTC arm compared with the DRV/r + RAL arm at week 96 (−3.8 vs +0.9 mL/min, respectively; *P* = 0.02). Creatinine clearance at 48 weeks in the A4001078 study [26,28,30] was stable with ATV/r + MVC therapy, but decreased with ATV/r + TDF/FTC therapy; at week 96, creatinine clearance decreased by 5.5 mL/min and 18 mL/min with ATV/r + MVC and ATV/r + TDF/FTC, respectively. In the SPARE study [42], there was no statistically significant difference between treatment groups in the number of patients who achieved a >10% improvement from baseline in eGFR with either DRV/r + RAL therapy or LPV/r + TDF/FTC. In ATLAS [45–47], eGFR was statistically increased versus baseline with ATV/r + 3TC (Cockroft-Gault equation, +6 mL/min, *P*<0.001 at 48 weeks; Modified Diet in Renal Disease equation, +15 mL/min, *P*<0.001 at 96 weeks). In the Reliquet 2014 study [52], serum creatinine improved in patients who switched to RAL + NVP therapy. The long-term clinical relevance of these statistical differences in renal parameters remains unexplored.

### Changes in Lipid Parameters

Changes in lipid parameters could serve as markers for future cardiovascular disease (CVD), an important issue in the aging populations of patients with HIV [9]. Along with traditional risk factors, such as smoking, some ARV agents have been associated with CVD [13,59–61]. Although NRTI-sparing dual therapy regimens may potentially improve lipid profiles and hence CVD risk, this question must be evaluated prospectively in the context of Framingham risk. Although the third agent included in triple therapy regimens may contribute to this risk, triple therapy regimens containing TDF tend to have more favourable lipid profiles compared with non-TDF regimens [18,19,21,23,24,34,36,37,44,50,62]. With the exception of the RADAR study (DRV/r + RAL), across the studies identified here, lipid abnormalities, especially elevations in triglyceride levels, were more frequent with dual therapy in CCTG 589, PROGRESS, KITE (all LPV/r + RAL), ACTG A5262 (DRV/r + RAL), ACTG 5142 and A5116 (both LPV/r + EFV) [18,19,21,23,24,36,37,44,50]. However, in the VEMAN (LPV/r + MVC) and SPARTAN (ATV + RAL) studies, the metabolic profile was found to be fairly stable between dual and triple therapy regimens [16,27,29]. In BMS-121 [35], the higher ATV dose was associated with a greater change in fasting triglycerides. The implications of these changes for CVD risk are uncertain. Interestingly, the positive impact of NVP on lipid profiles was maintained when used in combination with LPV/r [51]. Investigators postulated that the improved atherogenic profile of LPV/r + NVP therapy among ARV-experienced, virologically suppressed patients could help lower the risk of cardiovascular events [51]. In the Reliquet 2014 (RAL + NVP) and ROCnRAL ANRS157 (RAL + MVC) studies, ARV-experienced, virologically-suppressed patients experienced decreases in lipid parameters after the switch to dual therapy [52,54].

### Hyperbilirubinaemia

Hyperbilirubinemia is frequently reported with ATV therapy. In some NRTI-sparing regimens, the rate of hyperbilirubinemia is increased, potentially limiting the use of ATV in dual therapy regimens [16,26,30]. However, total bilirubin levels were not elevated by ATV used in combination with 3TC in the ATLAS trial [45,47].

### Bone Health

Osteoporosis is common in aging populations, especially in postmenopausal women. Thus, it is disconcerting that increased rates of BMD loss are observed in patients with HIV [63]. The relative contributions of HIV or its therapy to these observations remains uncertain. As has been noted in a number of recent studies, there is a decline in BMD of 2% to 6% with the initiation of ARV therapy [64]. This has been found to be more marked in those regimens in which TDF is included [65,66]. Because HIV treatment is continued over the long-term, the implications for bone health are worrisome. Few studies identified in this analysis examined the effect of dual therapy regimens on BMD.

PROGRESS [24] evaluated the change in BMD through 96 weeks and markers of bone turnover. The mean change from baseline in BMD was −2.48% for the LPV/r + TDF/FTC arm compared with an increase of 0.68% for the LPV/r + RAL arm (*P*<0.001). Bone turnover markers C-terminal telopeptide (CTx) and osteocalcin increased in both groups to week 96, with greater increases in the LPV/r + TDF/FTC arm. Early changes in bone turnover markers were associated with a >5% BMD decrease at week 96, and increases at 4 weeks in CTx were associated with clinically significant bone loss. TDF had a greater impact on bone turnover and was associated with a higher incidence of clinically significant bone loss compared with RAL in combination with LPV/r. Similar positive findings on bone markers were observed in ATLAS, where there was a trend toward increased lumbar spine vertebrae 2 and 4 BMD in the ATV/r + 3TC arm [47]. The ROCnRAL study [67] noted a mean increase in BMD during a 26-week evaluation period, corresponding to an estimated increase of 2% per year. Although these data provide evidence that measurement of bone markers may be predictive of changes in BMD and may be useful for biotherapeutic agent evaluation, caution is warranted because of the small number of study subjects, the less specific imaging used, and the wide variation in bone marker measures.

### Body Fat Redistribution

Lipoatrophy has frequently been associated with NRTIs; previous studies have shown that patients switched from zidovudine (AZT) and stavudine (d4T) experience improvements in peripheral fat. Therefore, a regimen that spares one or more NRTIs may improve or prevent lipoatrophy. However, compared with earlier NRTIs, the rate of lipoatrophy with newer NRTIs (eg, TDF and abacavir) appears to be low, making it unclear whether NRTI-sparing regimens will have much clinical impact. In the studies examined here, an increase in limb fat was observed in NRTI-sparing regimens compared with triple therapy regimens, both in ARV-naive and virologically suppressed patients [25,36,45,50].

## Discussion

A total of 29 studies that evaluated the effectiveness of novel dual therapy regimens were summarised in ARV-naive (16 studies) or ARV-experienced, virologically suppressed patients (13 studies). They provide preliminary, short-term insights into the potential of dual therapy regimens for achieving and maintaining adequate virologic suppression in these populations. Whether long-term viral suppression and reduced toxicity will be achieved and maintained remains unclear. Given the small patient numbers and short durations of most of the existing studies, it remains unknown to what extent resistance will develop upon failure of a particular regimen, or if re-suppression will be possible with a switch or intensification of therapy. Certain dual therapy combinations seem to have insufficient efficacy or safety profiles to be recommended as alternative options to current standard of care, specifically in ARV-naive patients: ATV 300 mg BID + RAL 400 mg BID due to high rates of severe bilirubinaemia [16], DRV/r 800/100 mg QD + RAL 400 mg BID in patients with CD4 counts <200 cells/μL [19], DRV/r 800/100 mg + MVC 150 mg QD due to inferior efficacy [31], and LPV/r 533/133 mg BID + EFV 600 mg QD due to poor tolerability [36,37]. Long-term studies evaluating efficacy, safety, adherence, pill burden, and cost-effectiveness are required to more completely understand the clinical value of other dual therapy approaches.

Because they were randomised and sufficiently powered to demonstrate noninferiority of the viral response at 48 weeks in the dual therapy arm compared with triple drug therapy comprising a PI combined with 2 NRTIs, the NEAT 001 study, which compared DRV/r + RAL with DRV/r + TDF/FTC, the PROGRESS study, which compared LPV/r + RAL with LPV/r + TDF/FTC, and the GARDEL study, which compared an ARV-sparing regimen of LPV/r +3TC with triple therapy, provide the most definitive evidence to date. NEAT 001 and PROGRESS assessed virologic efficacy up to 96 weeks [20,23,24], and all three studies found potential signals of reduced toxicity [23,24,34]. The GARDEL trial is further strengthened by the larger sample size, including a reasonable percentage of patients with a higher baseline viral load. To date, however, the findings of these studies have not been confirmed in second independent, adequately powered studies. In addition, no dual therapy regimens have been directly compared with each other in prospective, randomised controlled trials. As such, no definitive statement can be made regarding the dual therapy treatment strategy that would achieve the best virologic efficacy and safety outcomes in HIV-infected patients. In rare situations where NRTIs cannot be used owing to transmitted resistance or toxicity, the combinations of LPV/r + 3TC, LPV/r + RAL, or DRV + RAL could be considered as alternative options in treatment-naive patients.

Because some studies have suggested that TDF use is linked to renal injury [68,69] and an increased cardiovascular risk may be associated with ABC (although this remains controversial) [70–74], avoiding their use in certain patients may be prudent. According to current treatment guidelines, dual therapy with LPV/r + 3TC, LPV/r + RAL, or DRV + RAL can be considered when regimens containing ABC or TDF are not recommended or are contraindicated because of patient comorbidities, such as the presence of cardiovascular risk factors or pre-existing renal disease and a positive HLA-B*5701 test [2–4].

Carr et al [75] have cautioned that, although virologic noninferiority is an essential endpoint for simplification studies, it should not be the only endpoint, as virologic noninferiority alone is not a benefit. The disadvantages of existing ARV regimens, with respect to AEs, quality of life, cost, or other effects can only be addressed in a simplification study if the disadvantage is important, if the entry criteria are well-defined, and if there is adequate recruitment of at-risk participants to provide the statistical power that is required to yield clinically meaningful results. Furthermore, a clinically relevant endpoint is exponentially more meaningful than, for example, a statistically significant change in a laboratory parameter.

Unfortunately, most of the studies cited in this report have not adequately addressed the potential short- and long-term AEs with dual therapy regimens or demonstrated that the newer strategies provide important clinical benefits or are cost-effective in the long term. Taken as a whole, the potential benefits of dual therapy regimens for some organ systems (eg, kidney and bone) must be balanced against potential detriments in others (eg, CVD). Further, any negative effects of treatment may necessitate additional monitoring or treatment (eg, lipid-lowering agents). Finally, medication-related changes in lipids and other markers must be considered in the context of traditional risk factors on disease outcomes (ie, smoking) in aging HIV-positive populations [76,77]. This information will be critical before dual therapy regimens gain widespread use.

Implementation of a dual therapy regimen requires consideration of potential drug–drug interactions with other agents used as part of the ARV regimen, as well as other prescription and over-the-counter drugs. For example, because of effects mediated through cytochrome P450 metabolism, coadministration of NVP or MVC with certain PIs or other classes of drugs (eg, some NNRTIs, antifungals, antivirals, and antibiotics) can affect the plasma concentration of both agents; thus, coadministration may not be recommended or dose adjustments may be required [78,79]. Before initiation of an ARV regimen in the individual patient, evaluation of concomitant medication use and pertinent prescribing information, as well as other available resources [80], should be consulted to assess for any potential drug–drug interactions.

Limitations of this review include the restriction of eligible trials to those that evaluated dual therapy regimens in either ARV-naive or ARV-experienced, virologically suppressed patients. An examination of studies that assess the ability of dual therapy regimens to provide virologic suppression in ARV-experienced patients with VF (salvage therapy) [81,82] were beyond the scope of this report. Furthermore, patient subgroups, such as individuals with hepatitis C virus coinfection, were not examined, nor were study results stratified by age or gender. The effectiveness of dual therapy regimens in ARV-naive patients with baseline HIV-1 RNA levels >100,000 copies/mL is inadequately investigated in most of the studies reported. Therefore, careful interpretation of the findings is warranted. An evaluation of the effectiveness of dual therapy regimens in diverse patient population is also required. A potential concern with the use of dual therapy is the persistence of viral replication in reservoir sites such as the central nervous system (CNS) [83,84]. The evaluation of dual therapy regimens regarding penetration and effectiveness in potential reservoir sites, and specifically, CNS viral escape and the predictive value of CNS penetration effectiveness scores, requires further study.

Preliminary data from some studies are encouraging; however, it is not currently possible to recommend widespread adoption of novel dual therapy regimens. Future trials demonstrating adequate long-term efficacy and safety are required before dual therapy regimens can be incorporated into routine clinical practice for ARV-naive and ARV-experienced, virologically suppressed patients. Other dual therapy regimens that do not include a boosted PI are currently being investigated, and initial results show promise. For example, the exploratory proof-of-concept study using dolutegravir and lamivudine in ARV-naive subjects (PADDLE) showed no virologic failure in 20 patients after 24 weeks of therapy [85]. Another recent study conducted in ARV-experienced, virologically suppressed patients (LATTE) reported positive results using a dual regimen of an integrase inhibitor, cabotegravir, with rilpivirine [86]. Results from additional studies could lead to a shift in the treatment paradigm for HIV.

## Supporting Information

S1 PRISMA Checklist PRISMA checklist. (DOC)
